# Natural Alkaloids as Antiviral Agents Against RNA Viruses: A Comprehensive and Mechanistic Review

**DOI:** 10.3390/molecules31030539

**Published:** 2026-02-03

**Authors:** Kristi Leka, Lúcia Mamede, Elyn Vandeberg, Mutien-Marie Garigliany, Allison Ledoux

**Affiliations:** 1 Faculty of Medicine, University of Liège, CIRM, Building B36 Quartier Hôpital, 4000 Liege, Belgium; kristi.leka@uliege.be (K.L.); mmgarigliany@uliege.be (M.-M.G.); 2 Faculty of Veterinary Medicine, University of Liège, FARAH-INDEEP, Building B42 Quartier Vallée 2 Avenue de Cureghem 6, 4000 Liege, Belgium

**Keywords:** alkaloids, RNA viruses, broad-spectrum antivirals, mechanisms of action, quaternary alkaloids, pharmacokinetics and toxicity, antiviral drug discovery

## Abstract

RNA viruses pose a persistent global threat due to their high mutation rates, zoonotic potential, and rapid adaptability. Emergence events have risen steadily, as demonstrated by major outbreaks caused by Influenza A, Ebola, Zika, and Chikungunya viruses, followed by the coronavirus epidemics of Severe Acute Respiratory Syndrome coronavirus (SARS-CoV-1) and Middle East Respiratory Syndrome Coronavirus (MERS-CoV) and culminating in the COVID-19 pandemic. These characteristics frequently compromise the durability of existing vaccines and antiviral therapies, highlighting the urgent need for new antiviral agents. Alkaloids, a structurally diverse class of nitrogen-containing natural compounds, have gained attention for their ability to interfere with multiple stages of the viral life cycle, including entry, replication, protein synthesis, and host immune modulation. To our knowledge, this review compiles all currently reported alkaloids with antiviral activity against RNA viruses and summarizes their proposed mechanisms of action, distinguishing evidence from in vitro, in vivo, and in silico studies. Quaternary alkaloids are discussed separately because their permanent ionic charge enables distinctive interactions with membranes and host pathways. Although many findings are promising, clinical translation remains limited by incomplete mechanistic validation, scarce in vivo data, suboptimal bioavailability, narrow therapeutic windows, and inconsistent experimental methodologies. To advance the field, future research should prioritize RT-qPCR–based antiviral evaluation to accurately quantify viral replication, incorporate mechanistic assays to clarify modes of action, apply structure–activity relationship (SAR) approaches for rational optimization, and expand in vivo pharmacokinetic and efficacy studies to assess therapeutic feasibility. Overall, alkaloids represent a promising yet underdeveloped reservoir for next-generation antiviral discovery against rapidly evolving RNA viruses.

## 1. Introduction

The objective of this review is to comprehensively compile naturally occurring alkaloids reported to exhibit antiviral activity against RNA viruses, a group characterized by frequent emergence, high mutation rates, and substantial pandemic potential. Beyond cataloging these compounds, the review critically examines their proposed mechanisms of action in relation to the experimental approaches used to characterize antiviral activity. Because the strength and translational relevance of reported findings depend strongly on study design, it is essential to distinguish between predictive computational analyses, cell-based assays, and animal models. While in silico screening and early in vitro studies provide valuable insights for compound prioritization, mechanistic validation and in vivo evaluation are required to substantiate therapeutic potential. Accordingly, this review explicitly differentiates alkaloids supported by in silico, in vitro, and in vivo evidence and discusses computationally identified candidates in a dedicated section, with emphasis on their limitations and translational relevance.

To support this analysis, a comprehensive literature search was conducted between August 2025 and December 2025 to identify peer-reviewed studies reporting antiviral activity of natural alkaloids against RNA viruses. The search strategy combined broad academic literature databases and targeted keyword-based queries to ensure extensive coverage across virus families and experimental contexts. Search terms included combinations of “alkaloids”, “quaternary alkaloids”, “antiviral”, and “RNA viruses”, together with the names of individual RNA viruses searched separately, including SARS-CoV-1, SARS-CoV-2, MERS-CoV, influenza A virus (IAV), dengue virus (DENV), Zika virus (ZIKV), Ebola virus (EBOV), West Nile virus (WNV), Japanese encephalitis virus (JEV), chikungunya virus (CHIKV), respiratory syncytial virus (RSV), hepatitis C virus (HCV), foot-and-mouth disease virus (FMDV), and porcine epidemic diarrhea virus (PEDV). Searches were performed iteratively to capture studies spanning in silico, in vitro, and in vivo experimental approaches.

Only peer-reviewed articles published in English were considered. Studies were included if they reported antiviral activity, target engagement, or mechanistic effects of natural alkaloids against RNA viruses based on primary computational, cellular, or animal data. Reviews, editorials, patents, and studies lacking primary experimental or computational evidence were excluded, except when cited to provide contextual background. Data extraction focused on alkaloid identity, viral target or virus family, experimental model, and reported antiviral outcomes, with emphasis on quantitative parameters when available.

In contrast to previous reviews focusing primarily on compound listings or single virus families, the present review emphasizes mechanistic patterns across alkaloid classes, distinguishes classical from quaternary alkaloids, and critically evaluates antiviral evidence according to experimental level (in silico, in vitro, in vivo). Particular attention is given to translational bottlenecks, including toxicity, pharmacokinetics, and formulation challenges.

### Overview of RNA Viruses and Their Global Health Impact

Viruses are microscopic infectious agents incapable of independent replication, as they lack the cellular machinery required for reproduction [1]. To propagate, they must invade a host cell and exploit its biosynthetic systems for their own replication. In 1971, David Baltimore introduced a classification system for viruses based on the type and replication strategy of their genetic material [2]. Within this framework, RNA viruses are distinguished by their ribonucleic acid (RNA) genomes, which confer unique structural and functional characteristics distinct from those of desoxyribonucleic acid (DNA) viruses.

A defining feature of RNA viruses is their exceptionally high mutation rate, primarily attributed to the low fidelity of RNA-dependent RNA polymerases, the enzymes responsible for genome replication [3]. This intrinsic genetic instability enables RNA viruses to adapt rapidly to new hosts and environmental pressures, often resulting in immune evasion and the reduced efficacy of antiviral drugs.

Many human RNA viruses are zoonotic in origin, arising in animal reservoirs before crossing the barrier into human populations. The likelihood of such “spillover” events is increasing due to anthropogenic factors such as deforestation, urbanization, climate change, and intensified human–wildlife interactions [4]. Notable zoonotic RNA viruses include the Human Immunodeficiency Virus (HIV), Influenza A virus subtypes H1N1 and H5N1, Severe Acute Respiratory Syndrome coronavirus (SARS-CoV), Middle East Respiratory Syndrome coronavirus (MERS-CoV), and Severe Acute Respiratory Syndrome coronavirus 2 (SARS-CoV-2).

The COVID-19 pandemic, caused by SARS-CoV-2 virus, underscored not only the profound global health threat posed by RNA viruses but also the degree to which the world remains unprepared for such challenges. The pandemic demonstrated how rapidly RNA viruses can evolve and disseminate across populations by producing numerous variants within a remarkably short period. Each of the variants was characterized by distinct levels of transmissibility, virulence, and clinical presentation [5,6,7,8,9]. The emergence and global spread of these variants placed extraordinary long-term strain on healthcare systems and triggered extensive social, economic and public health disruptions.

When a novel RNA virus emerges in the human population, pre-existing immunity is typically absent, rendering communities highly susceptible to infection. The continuous co-evolution of RNA viruses and their hosts further complicates outbreak prediction and as such it delays the development of effective vaccines and therapeutic solutions [10]. In summary the high mutability, zoonotic potential, and rapid adaptability of RNA viruses underscore their significance as persistent and evolving threats to global health.

## 2. Virus Classification in the Genomic Era

The classification of viruses is a dynamic and continuously evolving process, shaped largely by advances in genomic sequencing technologies. Historically, viruses were categorized by phenotypic traits, host range, and associated diseases [11]. Today, the International Committee on Taxonomy of Viruses (ICTV) serves as the authoritative body for viral taxonomy [12]. Modern systems rely heavily on phylogenetics and genomic data, frequently structured within the Baltimore classification framework [11].

Metagenomic expansion has produced large datasets that challenge existing taxonomic frameworks, especially given recombination and reassortment [12]. Integrative approaches combining sequence identity analyses with phylogenetic methods, for example in *Betaflexiviridae* as described by Silva et al. (2022), have been proposed [13]. These methods aim to refine viral classification to better account for the diversity revealed by high-throughput sequencing [13].

Despite advances, the Baltimore system remains a pillar in organizing viruses by genome type and replication mode although minor revisions have reflected newly identified behaviors and genome structures [12].

Baltimore divides the viruses into the following groups:

- I: dsDNA (e.g., Herpes simplex virus)
- II: ssDNA → dsDNA before transcription (e.g., Parvovirus)
- III: dsRNA; mRNA transcribed from RNA genome (e.g., Rotavirus)
- IV: (+)ssRNA; genome functions as mRNA (e.g., Picornavirus, Coronavirus, Flavivirus)
- V: (−)ssRNA; mRNA transcribed from RNA genome (e.g., Rabies virus, Influenza virus, Ebola virus)
- VI: ssRNA with reverse transcriptase (e.g., HIV)
- VII: dsDNA with reverse transcriptase (e.g., Hepatitis B virus)

This review aims its focus on Groups III, IV, V, and VI concerning only the RNA viruses responsible for major outbreaks in recent decades, including Influenza A virus, MERS-CoV, SARS-CoV, HIV, Ebola virus, Zika virus, Japanese Encephalitis virus and SARS-CoV-2.

## 3. Alkaloid Biosynthetic Pathways and Structural Diversity

Plant alkaloids are structurally diverse nitrogen-containing compounds with broad biological activity and long medicinal use. Their complexity arises from enzyme-mediated modifications, glycosylation, acylation, reduction, oxidation, and methylation, which diversify structures and modulate bioactivity [14]. Cytochrome P450–mediated oxidations are particularly pivotal, enabling ring formation, cleavage, and expansion that greatly increase molecular diversity [15].

Advances in molecular biology and biotechnology have illuminated genes involved in alkaloid biosynthesis. For example, Kishimoto et al. 2016 used synthetic biology and microbial systems (*Escherichia coli*, *Saccharomyces cerevisiae*) to verify gene function, optimize production, and generate structural analogs, facilitating scalable pharmaceutical applications [15,16].

Obtaining useful amounts of alkaloids from plants often requires large quantities of raw material, considerable time, and specialized equipment to extract and identify each compound. Because of these demands, traditional isolation can be costly and environmentally taxing. Biosynthetic approaches, on the other hand, offer a more sustainable and practical alternative, allowing alkaloids to be produced without relying heavily on plant harvesting.

### 3.1. Classification of Alkaloids

Alkaloids are commonly classified by biosynthetic origin, emphasizing the presence and source of the nitrogen atom. Protoalkaloids are defined by the nitrogen outside the heterocyclic ring. On the contrary, for the true alkaloids the nitrogen is incorporated within a heterocyclic ring. Whereas for the pseudoalkaloids, the nitrogen is derived from non–amino acid precursors [17].

A structural classification based on the ring systems is also available into the following groups: quinoline, isoquinoline, pyrrole, pyrrolidine, pyridine, piperidine, pyrrolizidine, and indole-containing alkaloids [18].

### 3.2. Host Hijacking and Viral Translation

RNA viruses have evolved strategies to hijack host machinery for replication and protein synthesis. By manipulating ribosomes, they redirect translation toward viral proteins, often at the expense of host antiviral factors [19]. Some cleave key host factors or modulate PRR (pattern recognition receptor) activation, evading immune detection and suppression [20,21].

Because many RNA viruses lack a canonical 5′ cap and differ in other structural features, they employ non-canonical translation mechanisms to initiate protein synthesis, including internal ribosome entry, leaky scanning, and ribosomal frameshifting. Viral RNAs can also form circular structures that, similar to cellular mRNAs, recruit host initiation factors to drive protein production [22].

A major challenge for RNA viruses is coordinating translation and replication, which compete for shared host factors. To ensure efficient infection, viruses must precisely regulate these processes, maintaining a balance that allows genome replication to proceed while still producing the proteins required for virion assembly.

### 3.3. Alkaloid Antiviral Modes of Action

Alkaloids exhibit antiviral activity against RNA viruses and, in some cases, DNA viruses, demonstrating effects at multiple stages of the viral life cycle [23,24]. Their antiviral mechanisms include inhibition of viral entry, either by interfering with receptor binding or by inducing membrane perturbation. They may also suppress viral replication through direct inhibition of essential enzymes such as RNA-dependent RNA polymerase (RdRp) or viral proteases. Additionally, several alkaloids disrupt viral protein synthesis, further limiting viral proliferation.

Beyond direct antiviral effects, alkaloids can exert indirect activity by modulating immune responses, including the regulation of cytokines, reactive oxygen species (ROS), and interferons [25].

Examples that illustrate these mechanisms include 10-hydroxyusambarensine and cryptospirolepine, which show strong binding affinity in silico for multiple viral targets [26,27]. Isoquinoline alkaloids modulate key inflammatory pathways such as NF-κB and MAPK/ERK [28]. Meanwhile, compounds such as tetrandrine, oxymatrine, and berberine demonstrate potent immunomodulatory activity by suppressing excessive inflammation [29].

During the course of this review, clear distinctions emerged between the antiviral activities of general alkaloids and those of their specialized subclass, the quaternary alkaloids. These differences were not limited to their molecular structures, specifically the permanently charged quaternary ammonium group, but were also evident at the pharmacological level.

Because quaternary alkaloids carry a permanent positive charge, their limited membrane permeability often restricts their antiviral activity to early steps of the viral life cycle, such as viral attachment or entry. They often act through mechanisms such as membrane disruption, interference with viral entry, or modulation of lysosomal and host signaling pathways. In contrast, many non-quaternary alkaloids display activity across a broader range of viral processes, including replication, protein synthesis, and immune modulation.

Differences were also observed in potency, cytotoxicity, selectivity index, bioavailability, and overall potential for clinical translation. Given these mechanistic and pharmacological distinctions, we determined that it was more appropriate to present alkaloids and quaternary alkaloids in two separate tables (Table 1 and Table 2) accordingly to ensure clarity and scientific accuracy.

In this review, antiviral evidence is discussed according to the experimental level at which it was generated, namely in silico, in vitro, and in vivo. While computational studies provide valuable hypotheses regarding potential targets and binding modes, they do not constitute proof of antiviral efficacy and must be interpreted with caution. In vitro and in vivo studies are therefore discussed separately where possible to reflect their respective levels of biological validation.

**Figure 1:**
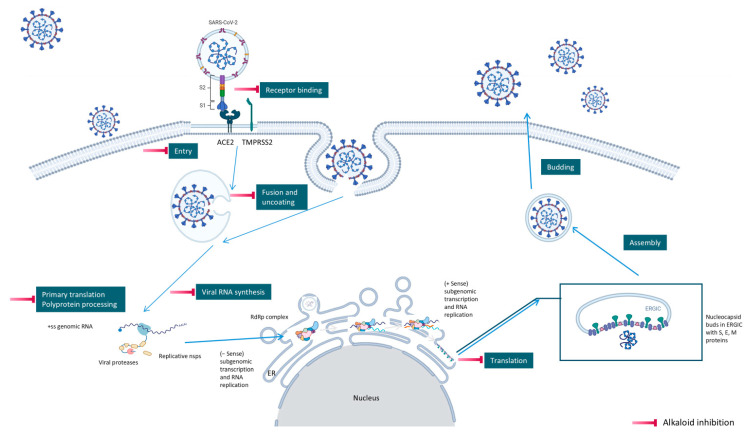
Overview of the SARS-CoV-2 replication cycle and the critical stages targeted by antiviral alkaloids.

Figure 1 presents SARS-CoV-2, the most recent coronavirus responsible for a global pandemic, as an example to illustrate the viral replication cycle within the host and the key steps at which a known alkaloid can exert inhibitory activity.

## 4. Quaternary Alkaloids: Distinct Mechanisms and Pharmacology

Quaternary alkaloids constitute a distinct subclass of alkaloids defined by a permanently charged quaternary ammonium group. Because of this ionic structure, they exhibit physiochemical and pharmacological properties distinguishing them from other alkaloid types.

Quaternary alkaloids can influence several stages of the viral replication cycle, including entry, replication, protein synthesis, and modulation of host responses, similar to alkaloids in general. However, because of their permanent positive charge, these compounds have low membrane permeability and limited intracellular diffusion. As a result, their antiviral activity is often strongest during the early steps of infection, particularly at the level of viral attachment or entry. For example, tetrandrine, a known quaternary alkaloid, blocks viral entry by targeting NPC1, causing lysosomal cholesterol accumulation and triggering interferon responses via NPC1–STING [110].

Unfortunately, quaternary alkaloids display poor bioavailability, rapid clearance, and short half-lives [111]. However, emerging delivery technologies such as liposomes, nanoparticles, transdermal systems can improve absorption, extend circulation, and enhance pharmacological performance [111,112,113]. Another important issue with the quaternary alkaloids is that the activity often occurs near the cytotoxic thresholds [114]. The following (Table 2) summarizes the key quaternary alkaloids studied to date and their antiviral activities across RNA viruses.

**Table 2:** Quaternary alkaloids displaying antiviral activity.

Compound & Origin	References	Study Type	Virus	Mechanisms	Antiviral Information Reported in the Original Studies (IC_50_, EC_50_, SI, Docking Scores, Qualitative Observations) *
*1.* ***Berberine and derivates*** (From: *Berberidaceae*/*Ranunculaceae* family) 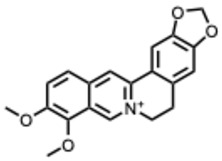	Wu et al. 2011 [115]	In vitro and in vivo	IAV	Berberine exerted strong inhibition on the inflammatory substances production	IC_50_ = 0.025 g/L Decrease in mice mortality from 90% to 55%
	Wang et al. 2018 [116]	In vitro	EV71	Might inhibit MEK/ERK, suppresses autophagy (AKT, JNK, PI3KIII)	IC_50_: 7.12–14.8 μM (Compound **2d**) and 7.43–10.25 μM (berberine)
	Shao et al. 2020 [117]	In vitro and in silico	HIV-1, clade B	It binds in the pocket of NHR and CHR of gp41.	IC_50_: 5.5–10.25 μg/mL
	Ratanakomol et al. 2021 [118]	In vitro	DENV, ZIKV, CHIKV	Potential AMPK activation, lipid metabolism disruption, direct virucidal activity	IC_50_: DENV: 42.87 μM; ZIKV: 11.42 μM; CHIKV: 14.21 μM
	Botwina et al. 2020 [119]	In vitro	IAV (H3N2)	Inhibits MAPK/ERK	IC_50_: MDCK = 52 μM; A549 = 17 μM; LET1 = 4 μM; Human airway epithelial (HAE) = 16 μM
	Enkhtaivan et al. 2017 [120]	In vitro and in silico	IAV	Competitive neuraminidase inhibition	Berberine derivatives IC_50_ H1N1: 0.87–1.63 µg/mL H3N2: 1.15–2.98 µg/mL
	Varghese et al. 2016 [121]	In vitro	CHIKV	Might be affecting one or several host factors important for CHIKV replication	IC_50_: 1.9 ± 0.9 μM
	Nguyen, C. Q. et al. (2021) [122]	In vitro and in silico	ZIKV	Potential candidate to inhibit NS2B-NS3 protease	Compound **4d**: Selectivity index (SI): 15.3 IC_50_ = 5.3 ± 1.9 µM
*2.* ***Chelerythrine*** (From: *Papaveraceae* family) 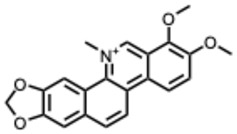	Españo, E. et al. (2022) [123]	In vitro, in silico	ZIKV	Potential entry/attachment inhibition	EC_50_ = 692.4 nM SI = 6.0
	Loe, M. et al. 2023 [124]	In vitro, in vivo	ZIKV	Potent inhibitor of ZIKV infection that targets the ZIKV NS4B protein	Chelerythrine chloride IC_50_ = 0.2513 µM. A 1.73 and 2.0 log 10 reduction in RNA copies/mL
	Guo, W. et al. 2020 [125]	In vitro (*plant*)	Tobacco Mosaic Virus (TMV)	Inactivation/proliferation inhibition	Chelerythrine at 0.5 mg/mL: 72.67% inactivation, (corresponding to 1.4 mM **)
*3.* ***Dehydrocorydaline*** (From: *Papaveraceae* family) 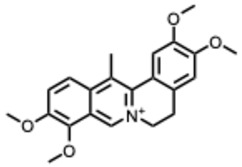	Orhan, I. et al. 2007 [126]	In vitro	Parainfluenza-3	N/A	CPE inhibitory concentration 16 µg/mL (≈ 40–44 µM ***)
*4.* ***Oxymatrine*** (From: *Fabaceae* family) 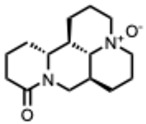	Dai, J. et al. 2018 [127]	In vitro and in vivo	IAV multiple strains (H1N1, H9N2, H5N1, H3N2)	Could significantly decrease the promoter activity of TLR signaling pathways TLR4, p38 MAPK, NF-κB	EC_50_: 5.91–23.67 µg/mL (strain-dependent) (= 22–90 µM *)
	Zhi et al. 2024 [128]	In vitro	H9N2 Avian Influenza Virus (AIV)	TLR signaling pathways TLR3, NF-κB, IRF-3	Dose-dependent effects on the cell survival rate
	Chen, N. et al. (2016) [129]	In vitro	HCV	Proliferation inhibition	Inhibition of cell proliferation: up to 85.4% at 72 h, 12mg/mL; mRNA expression 0.59 ± 0.12 vs. control
*5.* ***Oxysophoridine*** (From: *Fabaceae* family) 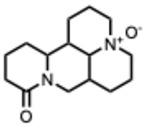	Majnooni, M. et al. (2021) [33]	*Review*	SARS-CoV-2	Nucleotide biosynthesis inhibitor	EC_50_ = 0.31 μM,
*6.* ***Palmatine*** (From: *Ranunculaceae*; *Rutaceae* family) 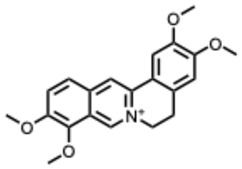	W. Zhang, 2024 [130]	In vitro	Infectious Bronchitis virus	Inactivating the virus, inhibiting its replication, modulating NF-κB/IRF7/JAK-STAT signaling pathways, and regulating apoptosis	IC_50_ = 7.76 µM Selection index (SI) was 86.74
	Fan Jia et al. 2010 [131]	In vitro	WNV	Palmatine could significantly inhibit the activity of NS2B-NS3 protease and that the inhibition was reversible	WNV: EC_50_: 3.6 µM, IC_50_: 96 µM
	Yi-Jung Ho et al. 2019 [132]	In vitro	ZIKV JEV	Inhibits Zika virus infection by disrupting virus binding, entry, and stability	Inhibited ZIKV binding by 95% and ZIKV entry by 69% Palmatine from 20–80 mM decreased JEV RNA levels.
*7.* ***Sanguinarine*** (From: *Papaveraceae* family) 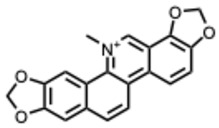	Qiyun Ke et al. 2023 [133]	In vitro and in silico	PRRSV	Targeting internalization, replication, and release stages of the viral life cycle	Sanguinarine inhibits the propagation of PRRSV in a dose-dependent manner

* When reported in µg/mL, IC_50_ or EC_50_ values were converted to µM using the molecular weight of the corresponding alkaloid when the chemical form was clearly specified. Values were left unconverted when compound form or purity was not explicitly stated. ** Converted assuming chelerythrine base; values were not converted when salt form was explicitly reported or not specified. *** Converted using the molecular weight of dehydrocorydaline (base) or dehydrocorydaline chloride when the salt form was specified; otherwise reported as an approximate range. In the transversal summary table, viral families are reported using commonly accepted abbreviated forms to preserve readability. To ensure taxonomic clarity, a legend has been added below the table explicitly indicating the corresponding ICTV family names (e.g., CoV = Coronaviridae, Flavi = Flaviviridae, Alpha = Alphaviridae).

To improve clarity and methodological transparency, we compiled an exhaustive transversal overview summarizing the level of experimental evidence supporting the antiviral activity of each alkaloid discussed in this review. Alkaloids are traditionally presented alphabetically to facilitate compound identification; however, this organization may obscure differences in experimental validation levels. The present table (Table 3) addresses this limitation by distinguishing in silico, in vitro, and in vivo evidence without duplicating compound-specific antiviral data already detailed in Table 1 and Table 2. Missing information is indicated by “–”, reflecting the absence of reported data rather than negative results. This overview highlights the current imbalance between extensive computational or cell-based screening and the limited number of compounds validated in vivo, underscoring key translational gaps in alkaloid-based antiviral research.

## 5. Discussion

Across both datasets, a coherent picture emerges, alkaloids represent a structurally diverse and mechanistically rich source of antiviral agents, with activity documented against a broad range of RNA viruses. Throughout this review, antiviral evidence is discussed according to the experimental level at which it was generated, namely in silico, in vitro, and in vivo, to reflect the degree of biological validation associated with each approach. In the first dataset, 42 compounds supported by 77 experimental entries show that approximately 45 percent of studies were performed exclusively in vitro, around 30 percent combined in vitro and in vivo approaches, and 25 percent relied mainly on in silico predictions. While computational studies provide valuable hypotheses and help prioritize candidate scaffolds, they do not constitute proof of antiviral efficacy and therefore require careful interpretation in the absence of experimental validation. This imbalance highlights extensive early screening but a shortage of deeper, mechanistic, and translational work. Another major bottleneck often occurs at the “in vitro–in vivo” transition, where a majority of compounds showing antiviral activity in vitro ultimately fail to demonstrate efficacy in vivo [23].

A central theme is the convergence of many alkaloids on viral replication machinery, particularly the RdRp. Compounds such as tetrahydroisoquinoline derivatives, lycorine, and aloperine consistently interact with RdRp or polymerase-associated subunits, often at low micromolar or nanomolar levels. Their rigid, polycyclic scaffolds appear intrinsically suited to binding nucleic acid–proximal enzymatic pockets, which may underlie their broad-spectrum effectiveness across coronaviruses, orthoflaviviruses, alphaviruses, and orthomyxoviruses.

A second mechanistic cluster involves entry inhibition. Neferine, fangchinoline, rutaecarpine, berbamine and capsaicin interfere with viral attachment, membrane fusion, or endosomal trafficking. These activities correlate with amphiphilic or cationic structural elements capable of perturbing lipid or acidic intracellular environments. Because many of these effects are host-directed, resistance may emerge more slowly, though this benefit must be balanced against increased toxicity risks.

Host-directed immunomodulation represents a third recurring pattern. Indirubin, emetine, ephedrine, and canthin-6-one derivatives modulate key pathways including NFκB, JAK, STAT, TLR, MAPK, and ERK. This dual antiviral and anti-inflammatory activity may be especially valuable in infections characterized by immunopathology such as influenza and SARS-CoV-2. At the same time, manipulating host signaling requires careful dosing and safety evaluation.

The second dataset, focused on berberine, chelerythrine, dehydrocorydaline, oxymatrine, palmatine, and sanguinarine, reveals similar mechanistic tendencies but with a clearer emphasis on innate immunity, inflammatory regulation, and endolysosomal physiology. Study distribution again skews toward in vitro work (≈50 percent), though about 35 percent include in vivo components. Berberine, palmatine, and oxymatrine frequently modulate TLR3, TLR4, NFκB, IRF3, AMPK, and autophagy-related pathways, producing broad-spectrum antiviral effects across influenza viruses, enteroviruses, flaviviruses, chikungunya virus, hepatitis C virus, and SARS-CoV-2. Meanwhile, quaternary or quaternary-like alkaloids such as chelerythrine and sanguinarine are particularly effective at disrupting viral entry and endosomal maturation through their permanent or semi-permanent cationic charge.

Cepharanthine (CEP) is one of the few alkaloids near clinical translation. Although an enhanced oral formulation (PD 001) received FDA clearance for Phase I and II testing in mild to moderate COVID 19, no PD 001 trials have begun, and the only active study is a Phase II placebo-controlled trial in mild COVID 19 (NCT05398705). Because intravenous CEP is impractical and standard oral dosing fails to reach antiviral levels, animal pharmacokinetic data indicating over 64 percent bioavailability with pulmonary delivery highlight inhaled formulations as the most promising route for clinical development [45].

Despite these strengths, significant limitations remain. Many alkaloids show narrow therapeutic windows, with active concentrations approaching cytotoxic levels. Depending on their chemical structure and administered dose, alkaloids may induce neurotoxicity, hepatotoxicity, nephrotoxicity, or cardiotoxicity [134]. Moreover, species-specific differences in metabolism and organ sensitivity further complicate the translation of preclinical findings to humans. A well-documented example is the class of pyrrolizidine alkaloids, which are strongly associated with hepatotoxic and carcinogenic effects due to their metabolic conversion into reactive intermediates [135]. Toxicity assessment is often limited to single- dose MTT assay (3-(4,5-dimethylthiazol-2-yl)-2,5-diphenyltetrazolium bromide assay) which measures cell metabolic activity or LDH assays (Lactate Dehydrogenase release assay) which measure cell membrane integrity in immortalized cell lines. However, these assays cannot predict organ-specific or long-term effects. Pharmacokinetic challenges such as poor oral bioavailability, limited solubility, extensive first-pass metabolism and short half-lives, further restrict their translational potential [136]. These issues highlight the need for optimized formulations, including nanoparticles, liposomes, prodrugs, and targeted delivery systems.

Beyond conventional molecular docking and virtual screening approaches, artificial intelligence (AI) and machine learning (ML)-based methods are increasingly being explored in antiviral drug discovery to prioritize natural compounds, including alkaloids. These approaches differ fundamentally from classical docking by integrating large multidimensional datasets to identify non-obvious structure–activity relationships through quantitative structure–activity relationship (QSAR) models, supervised learning algorithms, or deep neural networks. In principle, AI-driven screening may enable rapid prioritization of alkaloids with multi-target potential, optimization of scaffold–activity relationships, and early prediction of pharmacokinetic and ADMET properties. However, despite their growing visibility, the application of AI-based models to alkaloid antivirals against RNA viruses remains largely prospective. Model performance is strongly dependent on the quality, size, and chemical diversity of training datasets, which are often biased toward synthetic compounds and well-characterized viral targets. Moreover, AI predictions require experimental validation, as computational confidence does not necessarily translate into antiviral efficacy or acceptable toxicity profiles. At present, AI-based approaches should therefore be viewed as complementary hypothesis-generating tools rather than substitutes for experimental screening, and their integration into alkaloid-based antiviral research will depend on the generation of standardized, high-quality biological datasets.

In addition, natural source diversity remains underexplored. While terrestrial plants dominate the current research landscape, marine organisms and endophytic fungi represent rich reservoirs of chemically unique alkaloids featuring halogenation, sulfur bridges, or complex polycyclic structures [137,138]. Advances in metagenomics, genome mining, and synthetic biology offer powerful tools to identify and optimize these underused scaffolds, potentially unlocking new antiviral pharmacophores with mechanisms distinct from those found in traditional plant-derived compounds.

Complementary to these discovery-driven approaches, late-stage functionalization (LSF) strategies, including photochemical transformations, provide a powerful means to expand chemical diversity directly from complex natural alkaloid scaffolds. By enabling selective modification at advanced synthetic stages, LSF allows the generation of novel scaffold variants and unexplored chemical space that may not be accessible through biosynthesis or de novo synthesis alone, thereby broadening the pool of potential antiviral pharmacophores.

Resistance considerations also shape the translational landscape. Traditional antiviral development often suffers from the “one drug- one bug” limitation, where each compound is tailored to a single virus. Because many alkaloids act on conserved host pathways or multiple viral targets, they may avoid this bottleneck, reducing the likelihood of rapid resistance. Combination therapies represent another promising avenue [134]. Berberine’s ability to enhance antibiotic activity via efflux inhibition and immunomodulation suggests that pairing alkaloids with established antivirals could yield synergistic effects [139]. Still, such strategies require thorough pharmacokinetic and toxicological evaluation before advancing to clinical stages.

Major methodological and infrastructural challenges persist. Many RNA viruses of public health concern require high-containment biosafety laboratories, which remain limited globally. As a result, many studies rely on pseudoviruses or computational models rather than live virus assays. RT-qPCR is used to quantify viral RNA, but it does not measure infectious virus. Viral titration assays which directly quantify infectious particles remain the gold standard for assessing antiviral efficacy. Pharmacokinetic analyses and tissue distribution studies are rare, and in vivo experiments often rely on short-term endpoints rather than detailed immunological or virological profiling. More robust preclinical and clinical work is needed to establish dosing, optimize structures, and generate high-quality efficacy data [135,140].

Vaccination remains a central pillar of RNA virus control. Yet rapid antigenic evolution, as seen in influenza A virus and SARS-CoV-2, limits vaccine durability [141]. Newer mRNA and vector-based platforms improve adaptability, but they cannot replace the need for broad-spectrum antivirals. Alkaloids, with their diverse mechanisms and host-targeted effects, may serve as complementary tools alongside vaccination to strengthen preparedness for future outbreaks.

These findings highlight both the promise and the challenges of alkaloid-based antiviral development. Their structural diversity, multi-target mechanisms, and activity across unrelated RNA viruses make them compelling candidates for next-generation therapeutics. Yet meaningful progress will require deeper mechanistic validation, expanded in vivo research, improved pharmacokinetic and toxicity profiling, and better access to biosafety level laboratories infrastructure. By combining modern drug development strategies with the chemical richness of alkaloids, it may be possible to generate broad-spectrum antivirals capable of responding quickly and effectively to future RNA virus threats.

## 6. Conclusions

Overall, the evidence shows that alkaloids form a rich and versatile source of antiviral candidates, acting through multiple conserved mechanisms including RdRp inhibition, blockade of viral entry, disruption of glycoprotein maturation, and modulation of host immune pathways. Several compounds, such as lycorine, emetine, homoharringtonine, cepharanthine, berberine, and palmatine, demonstrate broad spectrum activity across diverse RNA viruses, suggesting that alkaloid scaffolds are well suited for addressing the rapid evolution and cross species transmission typical of these pathogens.

Yet significant gaps hinder clinical translation. Most studies depend on in vitro assays without confirming true viral suppression, in vivo evidence remains limited, and toxicity, bioavailability, and pharmacokinetic profiles are poorly characterized. Many alkaloids also have narrow therapeutic windows, and the lack of high containment biosafety facilities restricts more rigorous evaluation. To advance these compounds meaningfully, future research must prioritize standardized antiviral testing, comprehensive toxicology, improved delivery strategies, and exploration of combination therapies. With such efforts, alkaloids could evolve from promising laboratory findings into antivirals for future RNA virus threats.

## Figures and Tables

**Table 1:** Alkaloids displaying antiviral activity.

*Compound & Origin*	References	Study Type	Virus	Mechanisms	Values
**1.** ***1.2.3.4-Tetrahydroisoquinolines derivatives*** (From: e.g., *Papaveraceae* family) 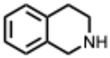	Y. Liao et al. 2023 [30]	In vitro, in silico and in vivo	IAV H1N1 H5N1 H3N2 Influenza B virus (IBV)	Mechanistic studies demonstrated that compound **35** could bind tightly to the PAN endonuclease of RNA-dependent RNA polymerase, thus blocking the viral replication to exert antiviral activity.	IC_50_ for compound **35** = 0.20 ± 0.01 µM EC_50_ = 0.88 µM SI = 113.1
	George, A. et al. (2018) [31]	In vitro	HIV-1	Inhibit the LEDGF/p75- IN	Compound **6d**: IC_50_ of ~10 μM
	Wang, X. et al. (2023) [32]	In vitro and in silico	SARS-CoV-2	Tt mainly inhibited the post-entry viral replication in both Vero E6 and Calu-3 cells.	Compound trans-1 EC_50_ = 2.78 µM SI > 71.94
**2.** **7-Methoxycryptopleurine** (From: *Menispermaceae* family) 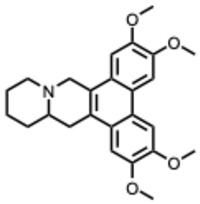	Majnooni, M. et al. (2001) [33]	*Review*	SARS-CoV-2	Blocking the S and N proteins, 3CLpro inhibitor	EC_50_ = 58 nM
**3.** ***10-Hydroxyusambarensine*** (From: *Strychnos usambarensis*, *Loganiaceae* family) 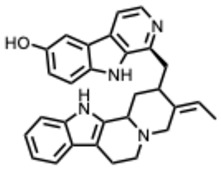	Ogunyemi, O.M. et al. (2020) [34]	In silico	SARS-CoV-2	Strong binding affinity to the RNA-dependent RNA polymerase (RdRp)	AutoDock version 4.2 programVina score: 10.1
**4.** ***Ajmaline*** (From: *Rauwolfia serpentina*, *Apocynaceae* family) 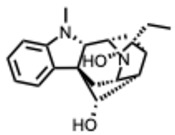	Cheng, F. et al. (2016) [35]	Computational biology	EBOV	Ajmaline predicted to up-regulates expression of several important Ebola-related genes, such as MERTK, FURIN, TYRO3, FURIN, and CTSB	q = 0.002
**5.** ***Aloperine and derivatives*** (From: *Sophora alopecuroides*/*Sophora flavescens*, *Fabaceae* family) 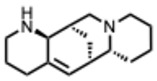	Cheng, F. et al. (2016) [35]	In vitro and in vivo	SARS-CoV-2	Inhibiting host cathepsin B activity and anti-cytokine effects	Compound **8a**: EC 50 = 39.1 µM SI > 6.8
	Zhou, P. et al. (2023) [36]	In vitro, in silico and in vivo	ZIKV	Targeting its RNA-dependent RNA polymerase (RdRp)	EC_50_: from 2.98–6.036 µM depending on the cells line SI: 31.72–66.95 depending on the cells line Aloperine administration resulted in an improved survival rate in mice and reduced viremia
**6.** ***Amarbellisine*** (From: *Amaryllidaceae* family)*** 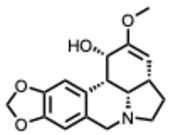 ***	Merindol, N. et al. (2024) [37]	In vitro	HCoV−OC43 HCoV-OC43	Potential inhibition of viral replication	SI = 60 EC_50_ = 0.2 µM
**7.** ***Berbamine*** (From: *Berberis* spp., *Berberidaceae* family) 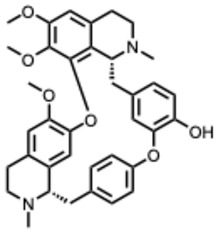	Huang, L. et al. (2021) [38]	In vitro	SARS-CoV-2	Compromising TRPMLs-mediated endolysosomal trafficking of ACE2	SARS-CoV-2: 2.35 ± 0.92 µM
**8.** ***Canthin-6-one and derivatives*** (From: *Rutaceae* family) 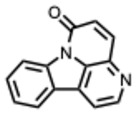	Wang, C. et al. (2024) [39]	In vitro	Newcastle disease virus (NDV)	Entry inhibition via Akt/ERK pathway; COX-2 induction	Analogue compounds: IC_50_ = 5.26–11.76 μM
	Verma, D. et al. (2020) [40]	In silico	SARS-CoV-2	Predicted binding to M^pro^ and PL^pro^, potential protease inhibition	Canthin-6-one 9-O-β-glucopyranoside PL^pro^: −9.4 kcal/mol M^pro^: −8.5 kcal/mol
**9.** ***Capsaicin*** (From: *Capsicum* spp., *Solanaceae* family) 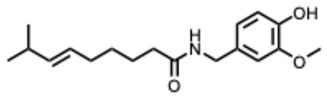	Zhang, M. et al. (2023) [41]	In vitro and in vivo	Encephalomyocarditis virus Vesicular stomatitis virus IAV H1N1	Capsaicin directly binds STAT3, promoting its lysosomal degradation	NA
	Marois, I. et al. (2014) [42]	In vitro	IAV	Capsaicin reduced influenza PA gene expression by ~48%	The EC_50_ was between 44.69–55.17 µM depending on the different strains of influenza virus
	Trischitta, P. et al. (2024) [43]	In vitro	Lassa Virus	Inhibits GP-mediated membrane fusion during viral entry	EC_50_ = 6.9–10.0 µmol/L
**10.** ***Cepharanthine*** (From: *Stephania* spp., *Menispermaceae* family) 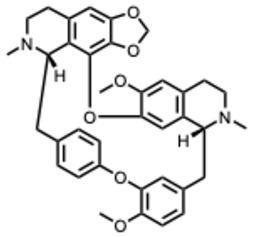	Xia, B. et al. (2023) [44]	Review	SARS-CoV-2	Inhibit viral entry and post-entry steps and attenuate the potential inflammatory effects	IC_50_ = 28.51 ng/mL
	Liu, K. et al. (2023) [45]	Review	1. SARS-CoV-2 2. SARS-CoV pseudovirus 3. MERS-CoV pseudovirus 4. HCoV-OC43 5. PEDV 6. SARS-CoV 7. EBOV 8. ZIKV 9. PRRSV 10. HIV-1	Multiple potential mechanisms of actions depending on the virus	1. EC_50_ = 0.15 M 2. EC_50_ ≈ 0.0417 µM 3. EC_50_ ≈ 0.14 µM 4. IC_50_ ≈ 0.83 µM 5. EC_50_ ≈ 2.53 µM; in vivo: 11.1 mg/kg oral dose reduced viral load 6. EC_50_ ≈ 0.79 µM 7. IC_50_ ≈ 0.42 µM 8. IC_50_ ≈ 2.19 µM 9. 10 µM reduced TCID_50_ ~5.6-fold; NF-κB inhibition 10. EC_50_ = 0.026 M
**11.** ***Cephaeline*** (From: *Cephaelis ipecacuanha*, *Rubiaceae* family) 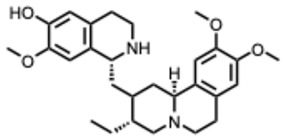	Ren, P. et al. (2022) [46]	In vitro and in silico	SARS-CoV-2	Targeting the host ribosome, and viral RNA, RdRp as well as N protein to interfere with the translating, propagating, replicating, and assembling process of the virus.	EC_50_ = 0.01 µM
	Yang, S. et al. (2018) [47]	In vitro, in silico and in vivo	ZIKV EBOV	Acts on the ZIKV RdRp NS5, host cell’s lysosome, and 40S ribosomal subunit	IC_50_ values of less than 42 nM IC_50_ = 16.9 nM with 95% CI of 10.7–25.8 nM
**12.** ***Cherylline*** (From: *Amaryllidaceae* family) 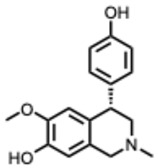	Ka, S. et al. (2021) [48]	In vitro and in silico	DENV ZIKV	Post-entry inhibition of RNA replication	EC_50_ = 8.8 µM SI = 28 EC_50_ = 20.3 µM SI = 12
**13.** ***Cinchonine*** (From: *Cinchona* spp., *Rubiaceae* family) 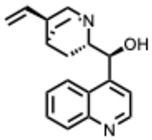	Ren, J. et al. (2022) [49]	In vitro	Porcine Epidemic Diarrhea Virus (PEDV)	Induction of autophagy, inhibiting early (adsorption/entry) and replication stages	Dose-dependent suppression of viral mRNA and N protein; ~100 µM nearly complete inhibition; RT-qPCR and TCID_50_ assays show significant reduction
**14.** ***Colchicine*** (From: *Colchicaceae* family) 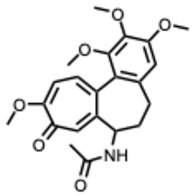	Hegazy, A. et al. (2024) [50]	In vitro and in silico	IAV H5N1 H1N1	Inhibition of viral adsorption and replication; docking supports NA/M2 binding	IC_50_ = 0.111 µg/mL IC_50_ = 0.326 µg/mL
**15.** ***Conessine*** (From: *Apocynaceae* family)*** 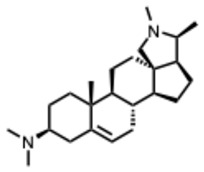 ***	Majnooni, M. et al. (2021) [33]	*Review*	SARS-CoV-2	M^pro^ inhibitor	EC_50_ = 2.34 μM,
**16.** ***Cyclopamine*** (From: *Melanthiaceae* family) 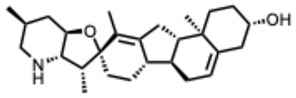	Bailly, C. et al. (2016) [51]	In vitro and In vivo	Human RSV (hRSV)	Disrupts inclusion bodies via M2-1 protein interaction, impairs RdRp complex	IC_50_ ≈ 380 nM; CC_50_ > 320 µM
	Diot, C. et al. (2023) [52]	In vitro and in silico	hRSV Bovine RSV (BRSV)	Hardens inclusion bodies, interferes with M2-1–P–RNA dynamics	Dose-dependent inhibition
	Fix, J. et al. (2023) [53]	In vitro	BRSV	Similar M2-1-mediated mechanism to hRSV	EC_50_ = 76 nM
**17.** ***Dehydroevodiamine*** (From: *Rutaceae* family) 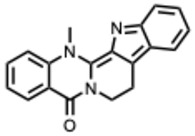	Li, K. et al. (2024) [54]	In vitro	PEDV	Inhibition of the PEDV replication stage, and its downregulation oft he ERK1/2 MAPH pathway	48 h PI: IC_50_ = 3.574 ± 0.566 µg/mL SI = 3.503
**18.** ***Deoxynojirimycin and derivatives*** (From: e.g., *Morus alba*, *Moraceae* family) 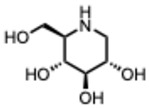	Hussain, M. et al. (2015) [55]	In vitro	IAV H3N2 strains	α-glucosidase inhibition, impaired viral glycoprotein folding	NN-DNJ IC_50_ = 0.5–2.5 µM depending on the viral strain
	Perera, N. et al. (2022) [56]	In vitro	DENV	Blocks viral release; inhibits glycoprotein maturation via α-glucosidase inhibition	IC_50_ of DNJ-iminosugars 48 h PI: 2THO-DNJ 1.6 ± 0.8 µM EOO-DNJ 3.1 ± 1.3 µM NN-DNJ 3.3 ± 1.5 µM
	Bhushan, G. et al. (2020) [57]	In vitro	ZIKV	Impairs viral replication by disrupting glycoprotein folding via ER glucosidase inhibition	At 1 µM, DNJ significantly reduced ZIKV RNA levels in supernatants compared to vehicle control (*p* ≤ 0.0318)
**19.** ***Emetine*** (From: *Rubiaceae* family) 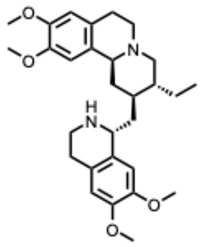	Yang, S. et al. (2018) [47]	In vitro and in vivo	ZIKV EBOV	Inhibits NS5 polymerase activity and disrupts lysosomal function; also inhibits EBOV entry	IC_50_ = 52.9 nM (95% CI: 35.4–73.2 nM) SJL mice; 1 mg/kg/day ~10-fold reduction in blood viremia at 7-day PI IC_50_ = 16.9 nM (95% CI:10.7–25.8 nM) 67% survival (4/6 mice) with IP emetine dosing
	Khandelwal, N. et al. (2017) [58]	In vitro and *in ovo*	PPRV NDV	Inhibits viral polymerase and entry; reduces viral RNA/protein synthesis	NDV: Reduced virus production 2-3 log in NDV infected cells
	Valipour, M. (2022) [59]	Review	SARS-CoV-2	Likely affects both virus- and host-based targets (translation machinery, NF-κB, etc.)	EC_50_ = 0.007 µM EC_50_ = 0.46 µM IC_50_ = 0.52 µM EC_50_ = 0.000147 µM EC_50_ = 0.00771 µM
	Bleasel, M. D. (2020) [60]	Commentary	SARS-CoV MERS-CoV	Broad-spectrum coronavirus inhibition suggested	EC_50_ SARS: 0.054 µM; EC_50_ MERS: 0.014 µM
**20.** ***Ephedrine and derivatives*** (From: *Ephedraceae* family) 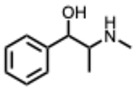	Wei, Y. et al. (2019) [61]	In vitro and in vivo	IAV (H1N1)	Modulation of TLR3/4/7 signaling, reducing TNF-α and increasing IFN-β	EC_50_ = 5.66–10.96 µg/mL depending on the drug delivery way
**21.** ***Fangchinoline*** (From: *Menispermaceae* family) 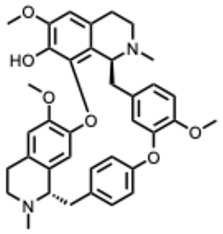	Yang, S. et al. (2024) [62]	In vitro and in vivo	ZIKA	Inhibits viral internalization	EC_50_: 0.86 ± 0.47 μM
	Wan, Z. et al. (2012) [63]	In vitro	HIV-1	Inhibits gp160 proteolytic processing, blocking envelope maturation	EC_50_: 0.8–1.7 µM depending on the HIV-1 strains
	Zhang, Q. Y. et al. (2024) [64]	In vitro	Enterovirus	Inhibits early-stage infection; VP1 mutations (E145G, V258I) reduce sensitivity	At concentration of 10 µmol/L FAN resulted in about 600-fold reduction in viral titers
**22.** ***Harmaline*** (From: *Nitrariaceae*/*Malpighiaceae* family) 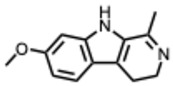	Hegazy, A. et al. (2023) [65]	In vitro	IAV H1N1 and H5N1	Not specified: measured via viral inhibition/cytopathic effect assay	H1N1: Harmaline: IC_50_ = 0.056 µg/mL H5N1: Harmaline: IC_50_ = 3.42 µg/mL
**23.** ***Harmine*** (From: *Nitrariaceae*/*Malpighiaceae* family) 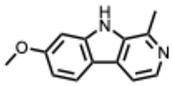	Hegazy, A. et al. (2023) [65]	In vitro	IAV H1N1 and H5N1	Not specified: measured via viral inhibition/cytopathic effect assay	H1N1: Harmine IC_50_ = 0.033 µg/mL H5N1: Harmine IC_50_ = 0.023 µg/mL
	Dahal, S. et al. (2023) [66]	In vitro	HCoV-229E SARS-CoV-2 variants HIV-1	Post-entry inhibition of viral replication via SR kinase inhibition; reduced viral protein expression and subgenomic RNAs.	Dose-dependent inhibition
**24.** ***Harringtonine*** (From: *Cephalotaxaceae* family) 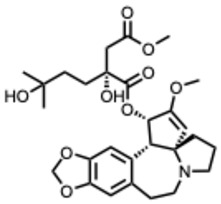	Kaur, P. et al. (2013) [67]	In vitro	CHIKV ZIKV	Inhibits viral protein synthesis by targeting host translation machinery	IC_50_ = 0.24 µM (plaque assay); EC_50_ = 0.29 µM (immunofluorescence assay)
	Lai, Z. et al. (2020) [68]	In vitro	ZIKV	Inhibits multiple stages: binding, entry, replication, release; also virucidal and prophylactic	~625 nM caused ~3-log reduction in viral RNA and titers
	Yang, Y. et al. (2023) [69]	In silico	SARS-CoV-2	Binds to spike RBD, TMPRSS2, and RBD–ACE2 complex to inhibit viral entry	High binding affinity in silico with RBD and TMPRSS2
**25.** ***Hernandezine*** (From: *Menispermaceae/Ranunculaceae* family) 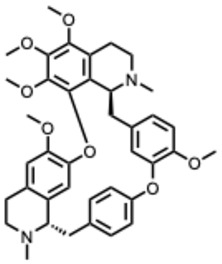	Majnooni, M. et al. (2021) [33]	*Review*	SARS-CoV-2	Blocking the calcium transition	EC_50_ = 10 μM,
**26.** ***Homoharringtonine*** (From: *Cephalotaxaceae* family) 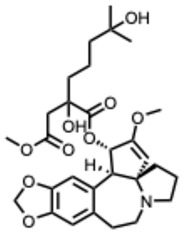	Dong. H. J. et al. (2018) [70]	In vitro	PEDV	Acts at early replication stages; additive effect with hydroxychloroquine (HCQ) or peptide tHR2	HHT (150 nM) reduced viral titers by ~3.5-fold; at 300 nM, approx. 40-fold reduction (TCID_50_ assay)
	Gong, M. et al. (2019) [71]	In vitro	Foot-and-mouth disease virus (FMDV)	Inhibits early stages of replication	Dose-dependent inhibition
	Harisha, K. R. et al. (2025) [72]	In vitro	Rabies virus (RABV)	Post-entry and antiviral across both lab-adapted and clinical isolates	EC_50_ ≈ 0.3 µM (BHK-21); EC_50_ ≈ 0.4 µM (Neuro-2a); cell–cell spread inhibited at ≈ 1.0 µM
	Neerukonda, S. N. et al. (2020) [73]	Review	SARS-CoV-2	Likely suppresses viral replication by targeting phosphorylated eIF4E and inhibiting host protein translation	EC_50_ ≈ 2.10 µM in Vero E6 cells
**27.** ***Indirubin*** (From: *Brassicaceae* family) 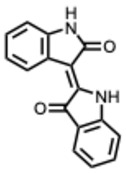	Mok, C. K. P. et al. (2014) [74]	In vitro	H5N1	Anti-inflammatory and antiviral via indirubin derivatives. Delays virus replication; reduces pro-inflammatory cytokines (IP-10)	Indirubin derivatives strongly suppress the pro-inflammatory cytokines including IP-10 (CXCL10), one of the key factors which contribute to the lung inflammation during H5N1 virus infection.
	Chang, S.-J. et al. (2012) [75]	In vitro and in vivo	JEV	Blocks viral attachment and has virucidal activity	EC_50_ = 0.006–0.105 mg/mL (concentration-dependent inhibition)
	Jie, C. et al. (2017) [76]	In vivo	IAV (H1N1)	Promotes MAVS-mediated IFN-β production and protects mitochondrial antiviral signaling.	Reduced lung NP levels, improved survival and lung pathology; enhanced IFN-β and IFITM3 signaling
	Medina-Moreno, S. et al. (2017) [77]	In vivo	HIV-1	Inhibits CDK9 to suppress HIV proviral transcription	Significant reduction in viremia at 5 mg/kg/day dosing
**28.** ***Lycorine and derivatives*** (From: *Amaryllidaceae* family) 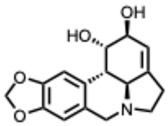	Jin, Y.-H. et al. (2021) [78]	In vitro	1. MERS-CoV	RdRp inhibition	IC_50_ = 1.406 ± 0.260 µM
		In vitro	2. SARS-CoV	RdRp inhibition	IC_50_ = 1.021 ± 0.025 µM
		In vitro	3. SARS-CoV-2	RdRp inhibition	IC_50_ = 0.878 ± 0.022 µM
	Chen, H. et al. (2020) [79]	In vitro and in vivo	ZIKV	Post-entry inhibition of RdRp activity	EC_50_ = 0.22–0.39 µM in different cell lines; CC_50_ = 4.4–21 µM; RdRp IC_50_ = 25 µM; 10 mg/kg in mice protected against lethality (~83%)
	Li, N. et al. (2021) [80]	In vitro	CHIKV	Inhibits viral translation post-entry	EC_50_ ≈ 10 µM
	Narayanan, A. et al. (2022) [81]	In vitro	SARS-CoV-2	M^pro^ inhibition; 88% reduction in viral spread in cell culture	EC_50_ = 0.01 µM (Lycorine HCl against M^pro^; SI = 1878)
	Fielding, B. C. et al. (2020) [82]	In vitro	SARS-CoV-2	Likely host-targeted antiviral modulation	EC_50_ = 300 nM; SI ≈ 130
**29.** ***Matrine and derivatives*** (From: *Fabaceae* family) 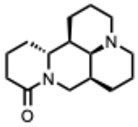	Pan, Q.-M. et al. (2015) [83]	In vitro	IAV H3N2	Inhibits viral replication	For Matrine type alkaloids: IC_50_ = 63.07–242.46 µM
	Qiao, W.-T. et al. (2024) [84]	In vivo	PEDV	Inhibits PEDV attachment and entry to cells.	EC_50_ = 0.09 µM (SI = 358.9)
**30.** ***Michellamine B*** (From: *Ancistrocladaceae* family) 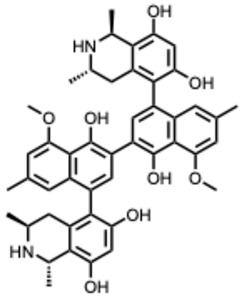	McMahon, J. B. et al. (1995) [85]	In vitro	HIV-1	Inhibits reverse transcriptase activity and viral-induced cellular fusion	EC_50_ = 1–20 µM Complete inhibition of cytopathic effects; RT enzymatic inhibition; IC_50_ ≈ 10 µM for fusion
**31.** ***Neferine*** (From: *Nelumbonaceae* family) 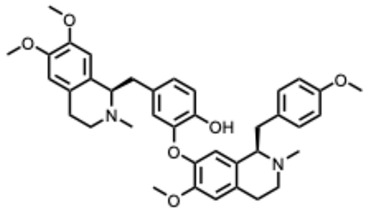	Yang, Y. et al. (2021) [86]	In vitro	SARS-CoV-2 Pseudovirus	Blocks host Ca^2+^-dependent membrane fusion → viral entry	EC_50_ = 0.13–0.41 µM
	Yang, D. et al. (2025) [87]	In vitro	SARS-CoV-2	Enhanced virucidal activity by salt form	Neferine free base: EC_50_ = 36.01 µM; Neferine salt: EC_50_ = 4.78 µM
**32.** ***Panicutine*** (From: *Ranunculaceae* family)*** 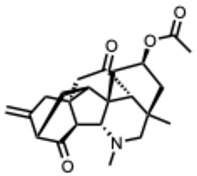 ***	Kumar, A et al. (2022) [87]	In silico	SARS-CoV-2	Potential M^pro^ inhibition	Binding energy: −7.4 kcal/mol
**33.** ***Piperine*** (From: *Piperaceae* family) 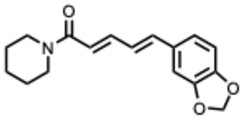	Nag, A. & Chowdhury, S. (2020) [88]	In silico	DENV EBOV	Potential inhibition of enzymatic targets	Stronger binding than ribavirin
	Pareek, A. et al. (2022) [89]	In vitro	CHIKV	RdRp inhibition, reduces viral replication	Kd = 0.08 µM (RdRp); EC_50_ = 6.68 µM
**34.** ***Pseudoephedrine*** (From: *Ephedraceae* family) 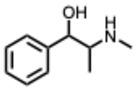	Deng, L et al. (2020) [90]	Review	IAV H1N1	Blunts cytokine storm, reduces lung inflammation, and inhibits virus replication	Increased life span in infected mice treated by PE
	Yu, S. et al. (2021) [91]	In vitro	SARS-CoV-2	Blocks viral entry and reduces inflammatory response	Inhibits spike pseudovirus entry; EC_50_ < 20 µM
**35.** ***Quinidine*** (From: *Rubiaceae* family) 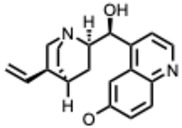	Yang et al. (2021) [92]	In vitro	SARS-CoV-2	Moderate antiviral activity among approved drugs in a high-throughput screen	IC_50_ = 0.42 µM (compound **6g**); IC_50_ = 1.41 µM (compound **7k**)
	Persoons, L. et al. (2021) [93]	In vitro	SARS-CoV-2 pseudovirus/HCoV-229E/OC43	Showed some broad-spectrum activity but only at relatively high doses	EC_50_ = 6 µM (SARS-CoV-2); EC_50_ = 0.2–9.4 µM (other HCoVs)
	Mamidala, E. et al. (2022) [94]	In silico	SARS-CoV-2 main protease (M^pro^)	Binds SARS-CoV-2 protease with predicted inhibitory affinity	Strong docking affinity
**36.** ***Quinine*** (From: *Rubiaceae* family) 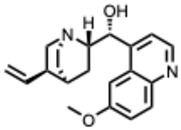	Malakar, S. et al. (2018) [95]	In vitro	DENV	Significant inhibition of DENV replication across multiple serotypes	~80% reduction vs. control
	D’Alessandro, S. et al. (2020) [96]	Review	IAV	Prophylactic plaque reduction at non-cytotoxic doses	NR
	Große, M. et al. (2021) [97]	In vitro	SARS-CoV-2	Complete viral inhibition at ≥50 µM; wider effective range depending on MOI and cell line	IC_50_ ≈ 25 µM (range: ~3.7–50 µM)
**37.** ***Rutaecarpine*** (From: *Rutaceae* family) 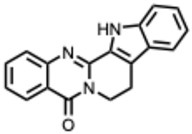	Lin, S. et al. (2023) [98]	In vitro and in silico	SARS-CoV-2 pseudovirus	Disrupts binding between spike protein and ACE2 receptor	IC_50_ ≈ 30 µM Omicron variant: IC_50_ ≈ 15 µM Binding energy ≈ −8.6 kJ/mol (wild-type), −10.2 kJ/mol (omicron)
**38.** ***Scopolamine*** (From: *Solanaceae* family) 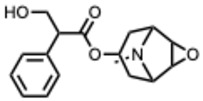	Bhattacharjee, A. et al. (2021) [99]	In ovo and in silico	JEV	Binds to the JEV NS5 protein and modulates TLR and IFN signaling pathways, potentiating antiviral innate immunity	Significant reduction in the viral load in CAM (*p* < 0.0001) and brain tissues (*p* < 0.0001) of the embryonated chick eggs when pre-treated with scopolamine hydrobromide
**39.** ***Sophocarpine*** (From: *Fabaceae* family) 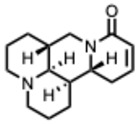	Jin, Z. et al. (2017) [100]	In vitro	Enterovirus 71 (E71)	Inhibits viral attachment, penetration, and RNA replication	IC_50_ = 350 µg/mL CC_50_ = 1346 µg/mL
**40.** ***Sophoridine*** (From: *Fabaceae* family) 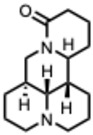	Ren, G. et al. (2019) [101]	In vitro	E71	Inhibits viral adsorption when added before infection	IC_50_ = 61.39 µg/mL
**41.** ***Strychnine*** (From: *Loganiaceae* family) 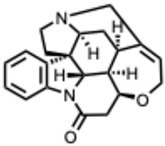	Hegazy, A. et al. (2023) [65]	In vitro	IAV H5N1 H1N1	Inhibits viral adsorption to host cells	IC_50_ = 11.85 µg/mL SI > 843 IC_50_ = 0.06 µg/mL; SI ≈ 167,000
**42.** ***Tetrahydropalmatine*** (From: *Papaveraceae* family) 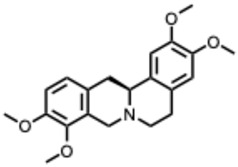	Lixia, H. et al. (2018) [102]	In vivo	JEV	Protects against neuronal apoptosis, reduces reactive oxygen/nitrogen species, lowers proinflammatory mediators, exhibits antiviral effects in brain tissues	Reduction in viral load in brain and CAM via neuroprotective and anti-inflammatory effects
**43.** ***Tetrandrine*** (From: *Menispermaceae* family) 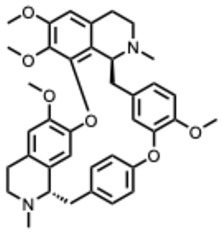	Kim, D. E. et al. (2019) [103]	In vitro	Human Coronavirus 0C43	Prevents viral replication and viral protein expression; effective mainly when administered pre- or during infection; also activates p38 MAPK signaling	IC_50_ ≈ 0.33 µM; SI > 40
	Liu, J. et al. (2023) [104]	In vitro and in vivo	SARS-CoV-2	Blocks viral entry at early stage by interfering with endosomal trafficking; exhibits favorable lung biodistribution when inhaled	EC_50_ = 0.40–5.03 µM depending on a cell line
**44.** ***Tylophorine and analogues*** (From: *Apocynaceae* family) 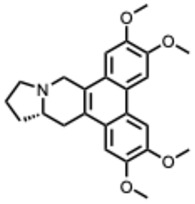	Fielding et al. (2020) [81]	In vitro	SARS-CoV	Blocks viral replication and cytopathic effects; potent inhibition of virus-induced apoptosis	EC_50_ = 5–340 nM (synthetic derivatives); 8–1468 nM (natural)
	T. I. M. et al. (2021) [105]	In vitro	SARS-CoV SARS-CoV-2	Inhibits viral RNA replication and NF-κB activation via JAK2 signaling pathway	IC_50_ = 58 nM (tylophorine), IC_50_ = 20 nM (7-methoxycryptopleurine)
	Yang et al. (2010) [106]	In vitro	Transmissible gastroenteritis virus (TGEV)	Targets viral RNA/RNP complex and inhibits NF-κB-mediated pro-inflammatory signaling	Substantial suppression of viral RNA replication (~4-log reduction)
	Wang, Y. et al. (2017) [107]	In vitro	Hepatitis C	Binds Hsc70 NBD, enhances ATPase activity → disrupts viral replication	Synthetic analogues of tylophorine DCB-3503 and rac-cryptopleurine EC_50_ = 30nM EC_50_ =300nM
**45.** ***Tryptanthrine*** (From: *Brassicaceae* family) 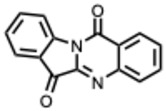	Mani, J. S. et al. (2020) [108]	Review	SARS-CoV-2 & Other HCoVs	Blocks coronaviral replication in early and late stages via inhibition of RdRp and papain-like protease	EC_50_ = 1.52 µM IC_50_ = 0.06 μM
**46.** ***Vilmorrianone*** (From: *Ranunculaceae* family)*** 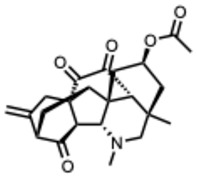 ***	Kumar, A et al. (2022) [87]	In silico	SARS-CoV-2	Potential M^pro^ inhibition	Binding energy: −7.0 kcal/mol
**47.** ***Vinblastine*** (From: *Apocynaceae* family) 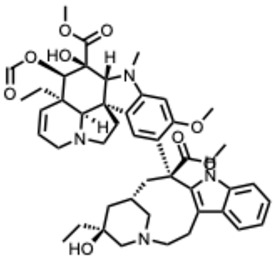	Akan, E. et al. (1997) [109]	In vitro	HIV-1	Vinblastine induced transcription through the HIV-1 long terminal repeat, suggesting modulation of NF-κB pathways, not antiviral action	~9–10-fold induction

When reported in µg/mL, IC_50_ or EC_50_ values were converted to µM using the molecular weight of the corresponding alkaloid when the chemical form was clearly specified. Values were left unconverted when compound form or purity was not explicitly stated. In the transversal summary table, viral families are reported using commonly accepted abbreviated forms to preserve readability. To ensure taxonomic clarity, a legend has been added below the table explicitly indicating the corresponding ICTV family names (e.g., CoV = Coronaviridae, Flavi = Flaviviridae, Alpha = Alphaviridae).

**Table 3:** Experimental Evidence Levels for Alkaloid Antivirals against RNA Viruses.

Alkaloid	In Silico	In Vitro	In Vivo	Virus families	Main Targets
**1,2,3,4-Tetrahydroisoquinoline derivatives**	✓	✓	✓	Orthomyxo, Retro, CoV	RdRp (PAN), IN
**7-Methoxycryptopleurine**	–	✓	–	CoV	Blocking the S and N proteins, 3CLpro inhibitor
**10-Hydroxyusambarensine**	✓	–	–	CoV	RdRp
**Ajmaline**	✓	–	–	Filo	Host gene regulation
**Aloperine**	–	✓	✓	Flavi, CoV	RdRp/Cathepsin B
**Amarbellisine**	–	✓	–	CoV	Potential inhibition of viral replication
**Berbamine**	–	✓	–	CoV	Endolysosomal trafficking
**Berberine**	✓	✓	✓	Orthomyxo, Flavi, Alpha, Retro	Host pathways/Entry
**Canthin-6-one derivatives**	✓	✓	–	Paramyxo, CoV	Proteases/Entry
**Capsaicin**	–	✓	✓	Orthomyxo, Picorna	STAT3/Host immunity
**Cepharanthine**	✓	✓	✓	CoV, Flavi, Alpha, Retro	Entry/Host pathways
**Cephaeline**	✓	✓	✓	CoV, Flavi	Ribosome/RdRp
**Cherylline**	✓	✓	–	Flavi	RdRp
**Cinchonine**	–	✓	–	CoV	Autophagy
**Colchicine**	✓	✓	–	Orthomyxo	Adsorption/Replication
**Conessine**	–	✓	–	CoV	M^pro^ inhibitor
**Cyclopamine**	–	✓	✓	Pneumoviridae	Inclusion bodies (M2-1)
**Dehydroevodiamine**	–	✓	–	CoV	ERK/MAPK
**Deoxynojirimycin derivatives**	–	✓	–	Orthomyxo, Flavi	α-glucosidase
**Emetine**	✓	✓	✓	CoV, Flavi, Filo, Retro	Translation/Entry
**Ephedrine**	–	✓	✓	Orthomyxo	TLR signaling
**Fangchinoline**	–	✓	✓	Flavi, Retro, Picorna	Viral internalization
**Harmaline**	–	✓	–	Orthomyxo	–
**Harmine**	–	✓	–	Orthomyxo, CoV, Retro	SR kinase
**Harringtonine**	✓	✓	–	Alpha, Flavi, CoV	Translation
**Hernandezine**	–	✓	–	CoV	Blocking calcium transition
**Homoharringtonine**	–	✓	✓	CoV, Flavi, Rhabdo	Translation
**Indirubin**	–	✓	✓	Orthomyxo, Flavi, Retro	MAVS/Cytokines
**Lycorine**	✓	✓	✓	CoV, Flavi, Alpha	RdRp
**Matrine**	–	✓	✓	Orthomyxo, Arteri	Viral entry
**Michellamine B**	–	✓	–	Retro	Reverse transcriptase
**Neferine**	–	✓	–	CoV	Ca^2+^-dependent fusion
**Oxysophoridine**	–	✓	–	CoV	Nucleotide biosynthesis inhibitor
**Panicutine**		✓			Protease (M^pro^)
**Piperine**	✓	✓	–	Flavi, Alpha	RdRp
**Pseudoephedrine**	–	✓	✓	Orthomyxo, CoV	Entry/Inflammation
**Quinidine**	✓	✓	–	CoV	Protease (M^pro^)
**Quinine**	–	✓	–	Flavi, CoV	–
**Rutaecarpine**	✓	✓	–	CoV	Spike–ACE2
**Scopolamine**	✓	✓	–	Flavi	NS5/Innate immunity
**Sophocarpine**	–	✓	–	Picorna	Viral attachment
**Sophoridine**	–	✓	–	Picorna	Viral adsorption
**Strychnine**	–	✓	–	Orthomyxo	Viral adsorption
**Tetrahydropalmatine**	–	–	✓	Flavi	Neuroprotection
**Tetrandrine**	✓	✓	✓	CoV, Orthomyxo	Entry/Endosomes
**Tylophorine & analogues**	–	✓	–	CoV, Alpha	RdRp/NF-κB
**Tryptanthrine**	–	✓	–	CoV	RdRp/PL^pro^
**Vilmorrianone**	✓	–	–	CoV	Protease (M^pro^)
**Vinblastine**	–	✓	–	Retro	Host transcription (No antiviral activity reported; host transcription modulation)

✓ = at least one primary study reported; – = no data reported in the reviewed literature. In the transversal summary table, viral families are reported using commonly accepted abbreviated forms to preserve readability. To ensure taxonomic clarity, a legend has been added below the table explicitly indicating the corresponding ICTV family names (e.g., CoV = Coronaviridae, Flavi = Flaviviridae, Alpha = Alphaviridae).

## Data Availability

No new data were created or analyzed in this study. Data sharing is not applicable to this article.

## Abbreviations

The following abbreviations are used in this manuscript:

ACE2	Angiotensin Converting Enzyme 2
ADMET	Absorption, Distribution, Metabolism, Excretion, and Toxicity
AIV	Avian Influenza Virus
AMPK	AMP-activated Protein Kinase
CAM	Chorioallantoic Membrane
CC_50_	50% Cytotoxic Concentration
CEB	Cepharanthine (context dependent, but in your text CEP is used)
CEP/PD-001	Cepharanthine/PharmaDrug oral formulation PD-001
CHIKV	Chikungunya Virus
CI	Confidence Interval
CPE	Cytopathic Effect
COVID-19	Coronavirus Disease 2019
CYP450	Cytochrome P450 Enzyme System
DENV	Dengue Virus
DNJ	Deoxynojirimycin
EC_50_	50% Effective Concentration
EBOV/Ebola	Ebola Virus
ELISA	Enzyme-Linked Immunosorbent Assay (appears implied in mechanistic work)
ER	Endoplasmic Reticulum
ERK	Extracellular Signal-Regulated Kinase
FDA	U.S. Food and Drug Administration
GP	Glycoprotein
HAE	Human Airway Epithelium
HBV	Hepatitis B Virus (if referenced)
HCV	Hepatitis C Virus
HCoV	Human Coronavirus
HIV	Human Immunodeficiency Virus
hRSV	Human Respiratory Syncytial Virus
IC_50_	50% Inhibitory Concentration
IFN	Interferon
IL	Interleukin
IRF	Interferon Regulatory Factor
JAK	Janus Kinase
JEV	Japanese Encephalitis Virus
JNK	c-Jun N-terminal Kinase
LDH	Lactate Dehydrogenase
LEDGF/p75	Lens Epithelium Derived Growth Factor
MAPK	Mitogen-Activated Protein Kinase
MERS-CoV	Middle East Respiratory Syndrome Coronavirus
MOI	Multiplicity of Infection
Mpro (3CLpro)	Main Protease of SARS-CoV-2
MTT	3-(4,5-Dimethylthiazol-2-yl)-2,5-Diphenyltetrazolium Bromide
NA	Neuraminidase
NBD	Nucleotide Binding Domain
NDV	Newcastle Disease Virus
NF-κB	Nuclear Factor kappa-light-chain-enhancer of activated B cells
NS proteins	Non-structural viral proteins (e.g., NS2B, NS3, NS4B, NS5)
PA	Polymerase Acidic Protein (Influenza)
PEDV	Porcine Epidemic Diarrhea Virus
PK	Pharmacokinetics
qPCR/RT-qPCR	Quantitative Real-Time Polymerase Chain Reaction
RdRp	RNA-dependent RNA Polymerase
RNP	Ribonucleoprotein
ROS	Reactive Oxygen Species
RSV/BRSV	Respiratory Syncytial Virus/Bovine RSV
SI	Selectivity Index
SARS-CoV-2	Severe Acute Respiratory Syndrome Coronavirus 2
TLR	Toll-Like Receptor
TMPRSS2	Transmembrane Protease Serine 2
TRPML	Transient Receptor Potential Mucolipin
TGEV	Transmissible Gastroenteritis Virus
US NLM	United States National Library of Medicine
VSV	Vesicular Stomatitis Virus
WNV	West Nile Virus
ZIKV	Zika Virus
